# Tumour-to-Tumour Metastasis: A Rare Case of Clear Cell Renal Cell Carcinoma Metastasizing Into Lung Adenocarcinoma

**DOI:** 10.1093/icvts/ivag201

**Published:** 2026-08-28

**Authors:** Valerio Sebastianelli, Silvia De Maria, Paolo Graziano, Marco Anile

**Affiliations:** Thoracic Surgery Unit, Department of General and Specialized Surgery and Anesthesiology, University of Rome Sapienza, 00161 Rome, Italy; Thoracic Surgery Unit, Department of General and Specialized Surgery and Anesthesiology, University of Rome Sapienza, 00161 Rome, Italy; Department of Radiological, Oncological and Pathological Sciences, University of Rome Sapienza, 00161 Rome, Italy; Department of Radiological, Oncological and Pathological Sciences, University of Rome Sapienza, 00161 Rome, Italy

**Keywords:** tumour-to-tumour, metastasis, lung cancer

## Abstract

Tumour-to-tumour metastasis (TTM) is a very rare condition in which a malignant tumour (donor) metastasizes into another primary neoplasm, either benign or malignant (recipient). We report the case of a 74-year-old man previously surgically treated for a clear cell renal cell carcinoma (ccRCC) who developed a new subsolid nodule in the right upper pulmonary lobe discovered at a 4-year follow-up CT scan. The patient underwent a right uniportal thoracoscopic S1 segmentectomy and mediastinal lymph node dissection. Histopathological examination revealed a lung adenocarcinoma (LUAD) containing a focus of metastatic ccRCC without lymph node involvement or evidence of additional metastatic disease. To date, the patient remains disease-free. Metastasis of renal tumour cells into a primary lung tumour is exceptionally rare. To our knowledge, this is the first reported case of ccRCC metastasizing into LUAD without evidence of systemic dissemination.

## BACKGROUND

Tumour-to-tumour metastasis (TTM) is a rare phenomenon whose underlying mechanism is not yet fully understood. It consists of a primary malignant tumour disseminating and growing within another benign or malignant tumour. Renal cell carcinoma represents one of the most frequent recipient neoplasms. Lung and breast cancers and melanoma are the most common donor malignancies. We report a case in which a tumour typically considered a “recipient” (clear cell renal cell carcinoma, ccRCC) metastasized into a tumour classically regarded as a “donor” (lung adenocarcinoma, LUAD), in the absence of other metastatic sites.

## CASE REPORT

A 74-year-old man who had undergone left radical nephrectomy for ccRCC in June 2021 (Fuhrman grade 2, pT1bN0, stage I) was referred to our department for a pulmonary nodule in the right upper pulmonary lobe. His medical history included systemic hypertension, and he was a former light smoker (1.25 pack-years).

In March 2025, a total-body CT scan identified a 12-mm spiculated nodule suggesting either an inflammatory lesion or a primary lung cancer. Owing to the technical difficulty of performing a CT-guided fine needle biopsy, our institutional multidisciplinary team decided on close radiological follow-up; a chest CT in June 2025 showed a slight enlargement of the lesion and a right uniportal thoracoscopic S1 segmentectomy with mediastinal lymph node dissection was performed.

Microscopic evaluation revealed invasive LUAD containing a microscopic focus of ccRCC.

Immunohistochemical analysis highlighted 2 distinctive tumour components demonstrating strong immunoreactivity for CD10 and PAX8 in ccRCC, whereas the LUAD component was positive for CK7 and TTF-1 only (**Figure 1**). LUAD exhibited a predominant acinar-papillary pattern with a solid/cribriform minor component and a peripheral non-mucinous lepidic pattern. Lymphovascular invasion was present and lymph node stations 4 and 10 R were negative. The patient was discharged on postoperative day 3.

**Figure 1.**
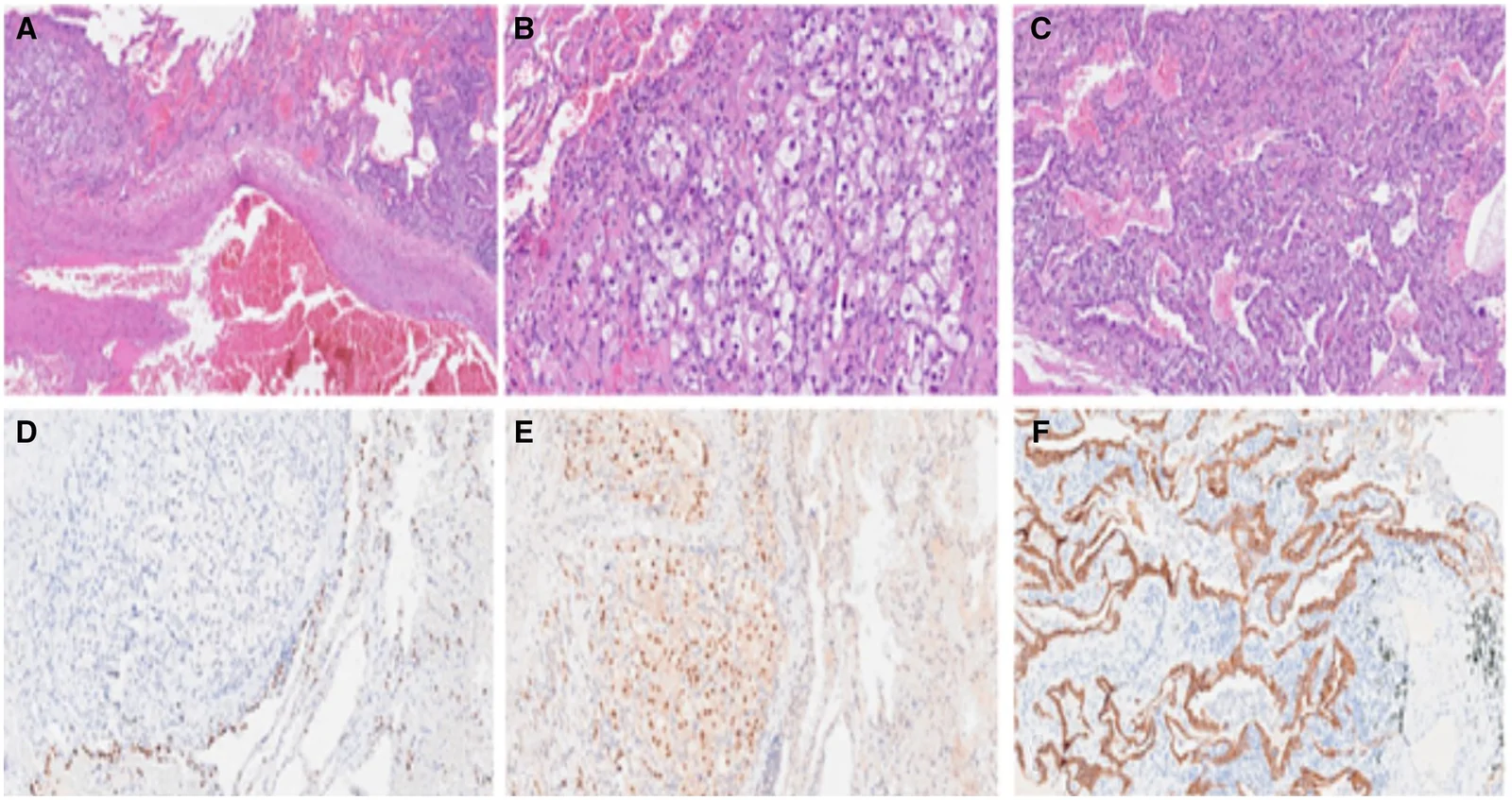
At Low Power Magnification (A, ×35 H&E) the Focus of Metastasis from ccRCC (Left Side) in the Context of a Non-Mucinous Lepidic Growth Pattern of Primary Invasive LUAD (Right Side). At higher magnification, morphological features of ccRCC and invasive LUAD, respectively (B and C, ×150 H&E) highlighted by negativity for TTF-1 (D, ×100) together with immunoreactivity for PAX8 (E, ×100) in ccRCC, whereas cytokeratin 7 (F, ×100) results positive only in LUAD

According to the UICC/AJCC2024 Classification, no adjuvant therapy was proposed and surgery was considered the definitive treatment with a R0 resection. A close radiological follow-up has been planned with a CT scan every 3 months for the first year and every 6 months for the next year; 9 months after the surgery the patient remains disease-free.

## DISCUSSION

TTM is the implantation and growth of metastatic tumour cells within a histologically distinct neoplasm, first described in 1902 by Berent.^1^ Campbell et al,^2^ proposed the diagnostic criteria to distinguish TTM from collision tumours, in which 2 different histologically distinct neoplasms are simultaneously present in the same lesion in the absence of a “donor” and a “recipient” tumour or direct tumour extension. Clinical history and careful histopathological examination, together with immunohistochemical and, when necessary, molecular analyses, are essential to differentiate between TTM and collision tumours. Our case fulfils all suggested criteria: (1) presence of 2 distinct primary tumours, (2) true neoplastic nature of the recipient tumour, (3) histologically proven growth of metastatic cells within the recipient, and (4) exclusion of tumour embolization or direct extension.

Several mechanisms have been proposed to explain TTM: (1) the mechanical theory in which an hypervascularized recipient tumour acts as a “trap” for circulating tumour cells, (2) the seed-and-soil hypothesis^3^ suggesting that certain tumour microenvironments provide a permissive niche for metastatic implantation, and (3) the role of the metabolic microenvironment: lipid-rich tumours show an abnormal lipid metabolism favouring the metastatic cells seeding.

A recent systematic review analysing almost 700 cases of TTM showed that RCC is among the most frequent recipient tumours.^4^ This predominance seems related to the enriched sinusoidal vascularization promoting the implantation of metastases, the high lipid and glycogen content modifying the tumour lipid metabolism creating a prometastatic niche and the favourable stromal microenvironment. Conversely, RCC is uncommonly a donor tumour. In our case LUAD, typically considered as a donor tumour, acted as a recipient for ccRCC cells. This form has been reported only twice previously (**Table 1**): the first was an autopsy finding reported by Campbell in 1968 in a patient with a primary hypernephroma with widespread metastases and a primary undifferentiated bronchogenic carcinoma harbouring metastatic foci of hypernephroma; in the other case, the patient had a current metastatic disease from RCC when he underwent a pulmonary resection for a LUAD with intratumoural metastasis from a papillary RCC.^5^ On the contrary, our patient showed a very limited and initial involvement by ccRCC cells without any signs of metastatic spreading.

**Table 1.** Published Cases of ccRCC Metastasizing to Pulmonary Neoplasms

Author, year	RCC histology	Recipient tumour	Location	Treatment	Outcome
Campbell 1968	Hypernephroma	Bronchogenic carcinoma	Right mainstem bronchus	N/A	Death
Caballero 2021	Papillary	Pulmonary adenocarcinoma	Left upper lobe	Lobectomy+systemic therapy	Stable disease

## CONCLUSION

This case expands the spectrum of tumour-to tumour metastasis and highlights an extremely rare biological interaction: ccRCC metastasizing into primary LUAD without evidence of systemic disease. Accurate histopathological evaluation and awareness of this phenomenon are essential for correct diagnosis and optimal clinical management.

## Data Availability

All data supporting the findings of this case report are included within the article.
